# Reduced Plasma Aβ Peptides but Stable NfL and GFAP in Major Depressive Disorder

**DOI:** 10.3390/ijms27031474

**Published:** 2026-02-02

**Authors:** María de los Ángeles Fernández-Ceballos, Lara Vidal-Nogueira, Carlos Fernández-Pereira, Pedro Fortes-González, Ángel Salgado-Barreira, Estrella Ledo-Matos, Elena Santana-Muriel, Tania Rivera-Baltanás, José Manuel Olivares, César Veiga, José M. Prieto-González, Roberto Carlos Agís-Balboa

**Affiliations:** 1 Translational Research in Neurological Diseases Group (ITEN), Health Research Institute of Santiago de Compostela (IDIS), Santiago University Complex, SERGAS-USC, 15706 Santiago de Compostela, Spain; maria.angeles.fernandez.ceballos@sergas.es (M.d.l.Á.F.-C.); lara.vidal.nogueira@sergas.es (L.V.-N.); maria.estrella.ledo.matos@sergas.es (E.L.-M.); roberto.carlos.agis.balboa@sergas.es (R.C.A.-B.); 2 Neurology Service, Santiago University Hospital Complex, 15706 Santiago de Compostela, Spain; elena.santana.muriel@sergas.es; 3 NeuroEpigenetics Lab., Health Research Institute of Santiago of Compostela (IDIS), Santiago University Hospital Complex, 15706 Santiago de Compostela, Spain; 4 Group of Genetics and Developmental Biology of Renal Disease, Laboratory of Nephrology, No. 11, Health Research Institute of Santiago de Compostela (IDIS), Clinical University Hospital of Santiago de Compostela (CHUS), 15706 Santiago de Compostela, Spain; pedro.fortes.gonzalez@sergas.es; 5 Department of Preventive Medicine and Public Health, University of Santiago de Compostela, 15782 Santiago de Compostela, Spain; angel.salgado.barreira@usc.es; 6 Health Research Institute of Santiago de Compostela (IDIS), 15706 Santiago de Compostela, Spain; 7 Consortium for Biomedical Research in Epidemiology and Public Health (CIBER en Epidemiología y Salud Pública-CIBERESP), Carlos III Health Institute, 28029 Madrid, Spain; 8 Translational Neuroscience Group, Galicia Sur Health Research Institute (IIS-Galicia Sur), Área Sanitaria de Vigo-Hospital Álvaro Cunqueiro, SERGAS-UVIGO, CIBERSAM-ISCII, 36213 Vigo, Spain; tania.rivera@iisgaliciasur.es (T.R.-B.); jose.manuel.olivares@gmail.com (J.M.O.); 9 Cardiovascular Research Group, Galicia Sur Health Research Institute (IIS Galicia Sur), 36213 Vigo, Spain; cesar.veiga@iisgaliciasur.es

**Keywords:** major depressive disorder, Alzheimer’s disease, biomarkers, amyloid beta-peptides, neurofilament proteins, glial fibrillary acidic protein, plasma, blood proteins, anhedonia, immunoassay

## Abstract

Major depressive disorder (MDD) has been associated with an increased risk of cognitive decline and neurodegenerative disorders like Alzheimer’s disease (AD), prompting interest in peripheral biomarkers related to amyloid metabolism as well as neuroaxonal and astroglial injury. However, evidence regarding circulating markers in MDD remains inconsistent. In this cross-sectional study, we simultaneously assessed plasma levels of amyloid-β peptides (Aβ40 and Aβ42), neurofilament light chain (NfL), and glial fibrillary acidic protein (GFAP) in MDD patients and healthy controls (HC) using ultrasensitive single-molecule array (SIMOA) technology. Associations with clinical and cognitive scales were examined. Plasma concentrations of Aβ40 and Aβ42 were significantly lower in MDD patients, whereas no group differences were observed for NfL and GFAP, after correcting for age and sex. However, both Aβ peptides were not significantly associated with depressive symptom severity, whereas the Aβ42/Aβ40 ratio was negatively associated with anhedonia. NfL and GFAP levels were primarily influenced by age. In the absence of a reduced Aβ42/Aβ40 ratio, these findings suggest that reduced plasma Aβ levels in MDD may reflect systemic or metabolic factors associated with MDD, including lifestyle or treatment-related effects. Therefore, these findings should be interpreted with caution and further examined in longitudinal studies to prevent potential confounding factors.

## 1. Introduction

Major depressive disorder (MDD) is one of the leading causes of disability worldwide, with a global estimation of 245 million cases [1]. Clinically, MDD is characterized by heterogeneous symptoms, with persistent low mood being the core feature. In addition, patients frequently experience fatigue, appetite and sleep disturbances, feelings of guilt, anhedonia, or abulia. Cognitive impairment may also be observed, and, in severe cases, MDD is associated with a markedly increased risk of suicide [2]. Therefore, MDD has increasingly been recognized as a mental condition with potential long-term consequences for brain health [3]

In this context, MDD has been postulated as a potential risk factor for neurodegenerative diseases, such as Alzheimer’s disease (AD) [4,5,6]. AD is characterized by progressive neuroaxonal damage, astroglial alterations, and deep dysregulation of amyloid-β metabolism [7,8,9]. These pathological processes have driven an intense search for biomarkers capable of capturing neurodegenerative changes at early stages, ideally through minimally invasive approaches. As a result, circulating biomarkers, such as neurofilament light chain (NfL), glial fibrillary acidic protein (GFAP), and amyloid-β peptides (Aβ40 and Aβ42), have emerged as peripheral indicators of these pathological processes in neurological diseases such as AD [10,11].

Amyloid-β (Aβ) peptides, particularly Aβ40 and Aβ42, are proteolytic products of the amyloid precursor protein (APP) generated through sequential cleavage by β- and γ-secretases [12]. In AD, alterations in amyloid-β metabolism provoke an imbalance between production and clearance, resulting in preferential accumulation of Aβ42 and deposition of amyloid plaques in the brain. In peripheral blood, absolute concentrations of Aβ peptides show substantial interindividual variability; therefore, the Aβ42/Aβ40 ratio has emerged as a more robust indicator of cerebral amyloid pathology, as it better reflects central amyloid burden while minimizing preanalytical and biological confounders [9].

Neurofilament light chain (NfL) is a neuron-specific cytoskeletal protein that constitutes a major structural component of large, myelinated axons and plays a vital role in axonal calibration and structural stability. Under physiological conditions, neurofilaments are highly stable and show slow turnover; however, disruption of neuroaxonal integrity leads to the release of NfL into cerebrospinal fluid (CSF) [13]. NfL crosses the blood–brain barrier (BBB) and reaches peripheral blood but at considerably lower concentrations, being a good estimation of central levels. Therefore, peripheral elevated circulating NfL levels are considered a sensitive marker of neuroaxonal injury, reflecting the extent of neuronal damage independently of the underlying diagnosis [14]. Murine depressive models have shown evidence of a significant reduction in NfL immunoreactivity in hippocampal neurons, suggesting cytoskeletal alterations [15,16]. However, NfL alterations may persist despite symptomatic improvement [17].

Glial fibrillary acidic protein (GFAP) is an intermediate filament protein predominantly expressed in astrocytes, where it plays a central role in maintaining astrocytic structure and cytoskeletal integrity [16]. In the context of brain pathology, astrocytes undergo reactive changes characterized by hypertrophy, proliferation, and upregulation of GFAP expression [18]. Increased levels of GFAP in CSF and peripheral blood have been proposed as indicators of astrocytic reactivity, reflecting glial responses to neurodegeneration or neuroinflammation [19]. Experimental data on rat models and post-mortem studies in humans suggest that MDD is primarily associated with astroglial atrophy or dysfunction, often reflected by reduced GFAP expression at brain levels rather than classical reactive astrogliosis [20,21,22].

Initially, studies evaluating NfL, GFAP, and Aβ levels in MDD primarily focused on CSF. Notably, one of the earliest studies reported higher CSF NfL levels in elderly women with MDD compared to healthy controls, whereas no significant differences were observed for GFAP or Aβ42 after adjustment for age [23]. Moreover, CSF NfL levels were found to be significantly increased in neurodegenerative disorders, such as AD or dementia, in contrast to psychiatric disorder, but the latter were not different from controls [24]. However, lumbar puncture is an invasive procedure, costly for healthcare systems, and requires specialized expertise, limiting its applicability in psychiatric populations. Consequently, increasing attention has been directed toward the analysis of peripheral fluids, such as blood (plasma or serum), which represents a less invasive, more accessible, and more cost-effective alternative.

Until recently, the study of peripheral biomarkers in MDD was hampered by limited analytical sensitivity, as circulating concentrations of neurodegeneration- and glia-related markers typically fall within the pg/mL range. While conventional immunoassays, such as ELISA, can detect marked elevations in neurological disorders [25], subtle alterations potentially present in primary psychiatric conditions like MDD, schizophrenia, or bipolar disorder remained difficult to capture and compare against a healthy control cohort. This limitation has been largely overcome by the advent of ultrasensitive single-molecule array (SIMOA) technology, enabling the reliable quantification of low-abundance biomarkers in peripheral blood [26,27].

Early peripheral studies in psychiatry predominantly focused on NfL, often aiming to differentiate primary psychiatric disorders from neurodegenerative conditions [28], finding that NfL levels were again significantly elevated in patients with neurodegenerative disorders in contrast to primary psychiatric disorders [29]. However, age has consistently emerged as a major covariate of peripheral NfL and GFAP concentrations in healthy populations, underscoring the need for careful adjustment in clinical studies. In this context, a recent Spanish study has suggested relevant age thresholds at 45 and 55 years for interpreting NfL and GFAP levels in peripheral blood [30], further highlighting the importance of age as a key confounding variables in these markers. 

Therefore, the interpretation of circulating biomarkers as surrogates of central nervous system pathology remains challenging. Peripheral blood concentrations of brain-derived proteins reflect the net result of limited brain-to-blood transfer, peripheral production, and multiple clearance mechanisms, rather than direct central release. Factors such as age, body mass index (BMI), pharmacological treatment, blood–brain barrier (BBB) transport, peripheral metabolism, cellular uptake by blood components, and systemic physiological variables may substantially influence plasma levels [28,31,32]. Consequently, alterations in circulating biomarker concentrations do not necessarily mirror corresponding changes at the central level, especially in non-neurodegenerative conditions, such as primary psychiatric disorders. This limitation is particularly relevant when studying MDD, where subtle biological alterations may coexist with substantial peripheral confounding.

The aim of the present study was to simultaneously assess plasma levels of NfL, GFAP, and amyloid-β peptides (Aβ40 and Aβ42) in patients with a diagnosis of major depressive disorder and compare them with a healthy control group. Beyond cross-sectional comparisons, we examined associations with clinically relevant variables, including age and sex, depressive symptom severity assessed using the Hamilton Depression Rating Scale (HDRS), anhedonia evaluated using the Self-Assessment Anhedonia State (SAAS) across its three domains (intensity, frequency, and change), and cognitive performance measured using the Mini-Mental State Examination (MMSE) and the Free and Cued Selective Reminding Test (FCSRT). We hypothesized that plasma amyloid-β peptides may show alterations in patients with major depressive disorder, whereas markers of neuroaxonal (NfL) and astroglial (GFAP) injury may not exhibit MDD-specific changes and would be more strongly influenced by demographic factors, such as age and sex. This hypothesis was explored within the context of a cross-sectional design.

## 2. Results

### 2.1. Plasma Levels of NfL, GFAP, Aβ40, and Aβ42 Between MDD Patients and HC Controls

First, we conducted a statistical comparison between MDD patients and HC controls for each marker (Table 1). Both NfL and GFAP plasma levels were found to be non-significant, whereas Aβ40 and Aβ42 were significantly reduced in MDD patients compared to HC controls (Table 1). However, MDD patients were found to be significantly older than HC controls, and sex was also differentially distributed between both groups (Table 1).

### 2.2. Age and Sex Distribution Correlations with Plasma Levels of NfL, GFAP, Aβ40, and Aβ42

**Figure 1:**
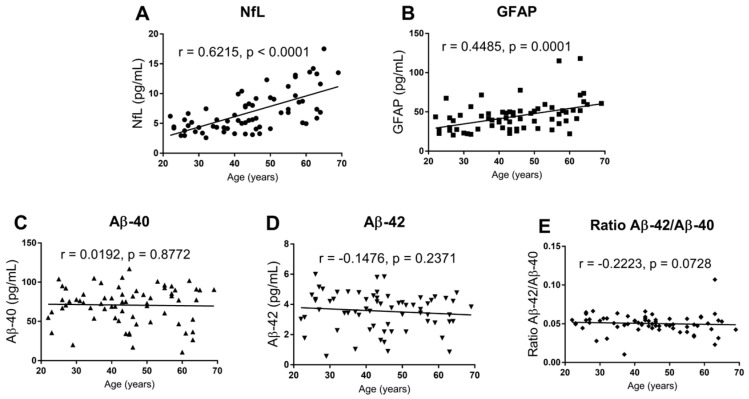
Associations between age and circulating biomarkers. Scatter plots showing the relationships between age and plasma levels of circulating biomarkers, including (**A**) NfL, (**B**) GFAP, (**C**) Aβ40, (**D**) Aβ42, and (**E**) ratio of Aβ42/Aβ40. Individual data points represent single participants. Spearman’s rank correlation coefficients (r) and corresponding *p*-values are indicated in each panel. Solid lines represent linear trend lines included for visual guidance only. NfL: neurofilament light chain, GFAP: glial fibrillary acidic protein, Aβ40: amyloid-β 1–40, Aβ42: amyloid-β 1–42. Ratio: calculated as the direct division of Aβ42 by Aβ40 levels.

Moreover, age was found to be significantly correlated with both NfL and GFAP levels, whereas this was not the case for both Aβ40 and Aβ42 or the ratio of Aβ40/Aβ42 (Figure 1).

**Figure 2:**
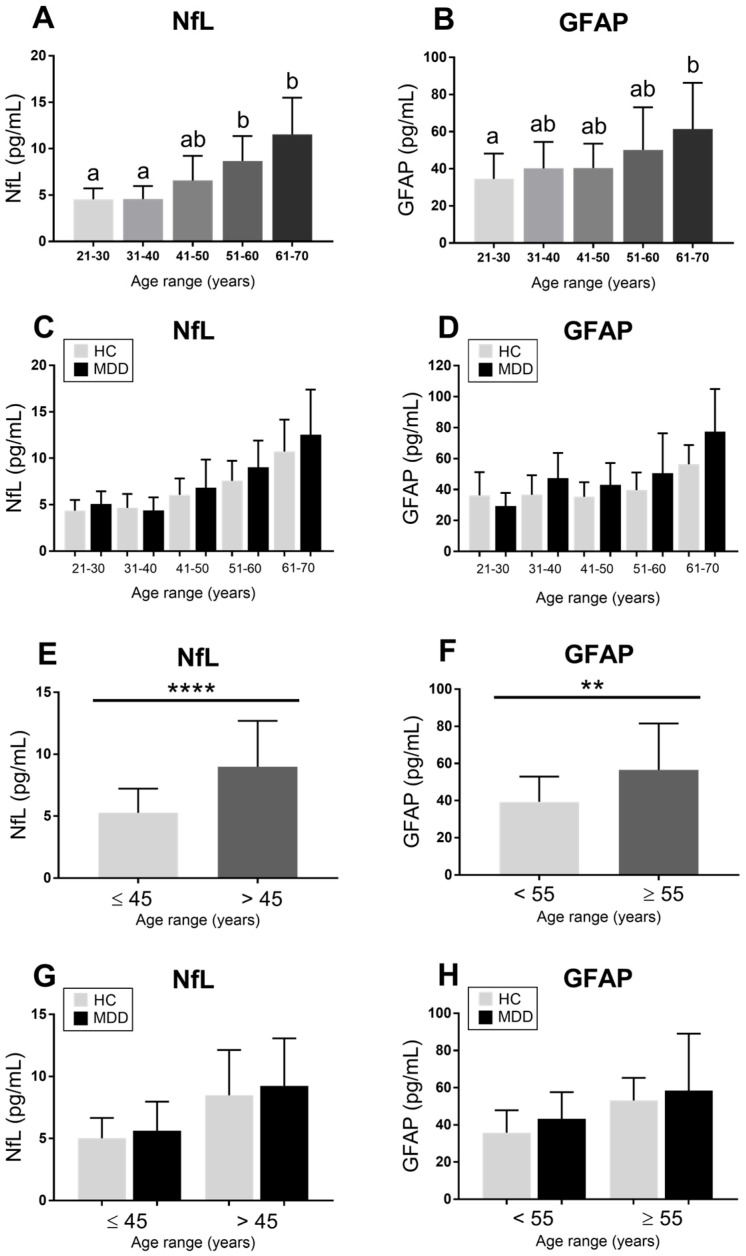
**Age-related differences in circulating NfL and GFAP levels**. Plasma concentrations of neurofilament light chain (NfL) and glial fibrillary acidic protein (GFAP) were analyzed across different age ranges. (**A**,**B**) show NfL and GFAP levels stratified by age groups (21–30, 31–40, 41–50, 51–60, and 61–70 years). Different letters indicate statistically significant differences between age groups based on the Kruskal–Wallis test followed by Dunn’s post hoc correction (*p* < 0.05). (**C**,**D**) show levels by age range and group (HC vs. MDD). (**E**,**F**) show age cut-offs. (**G**,**H**) are NfL and GFAP levels stratified by group within age categories. Statistical significance is indicated as follows: ** *p* < 0.01; **** *p* < 0.0001. Data are presented as mean values with standard deviation.

When stratifying all participants by age ranges, a graduate increase can be seen for NfL. Conversely, GFAP only reached significantly higher levels in the older group when compared to the youngest (Figure 2A,B). Nonetheless, when dividing by diagnostic group, no significant differences were found for each marker at each range (Figure 2C,D). Curiously, NfL and GFAP plasma levels were found to be significantly increased at the age cut-offs of 45 and 55 years in the whole sample (Figure 2E,F). However, when stratifying again for diagnostic group and age cut-off, only a slight increase without statistical meaning was found for each marker in the MDD group (Figure 2G,H).

**Figure 3:**
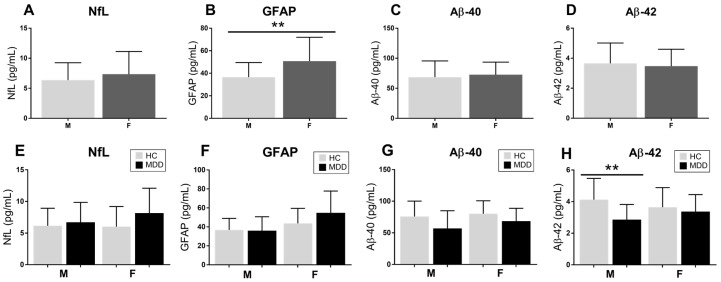
**Sex-related differences in circulating markers in the whole sample and stratified by diagnostic group.** First, we compared between male (M) and female (F) in the whole sample the plasma concentrations of (**A**) NfL, (**B**) GFAP, (**C**) Aβ40, and (**D**) Aβ42. Second, we stratified by diagnostic category, including healthy controls (HC) and patients with major depressive disorder (MDD), to compare (**E**) NfL, (**F**) GFAP, (**G**) Aβ40, and (**H**) Aβ42. Upper panels show sex-based comparisons across the entire cohort (**A**–**D**), whereas lower panels depict the same comparisons separately within HC and MDD groups (**E**–**H**). Statistical analysis was performed using the Mann–Whitney U test. Double asterisks indicate statistically significant differences between sexes (** *p* < 0.01). Data are presented as mean values, with standard deviation as error bars.

Detailed data from Figure 2 are available in the Supplementary Material in both Tables S1 and S2. Regarding sex distribution as a potential confounding factor, women showed a significant increase only in GFAP levels in contrast to men (Figure 3B), while no other significant difference was found for other markers (Figure 3A,C,D). Curiously, after separating by diagnostic group, men from the HC group showed significantly higher Aβ-42 plasma levels than MDD male patients (Figure 3H), whereas no other significant alteration was found regarding diagnostic group and sex distribution (Figure 3E–G). Expanded data can be consulted in the Supplementary Material in Table S3.

### 2.3. Multivariable Logistic Regression Models for Age and Sex Distribution

Therefore, we then conducted a multivariable logistic regression analysis controlling for age and sex, finding that both NfL and GFAP were not significantly associated with the MDD group, while Aβ40 and Aβ42 were independently associated with the diagnostic group. Specifically, lower Aβ40 levels were linked to an increased likelihood of belonging to the MDD group (Exp(B) = 0.967; 95% CI: 0.941–0.993; *p* = 0.015). Similarly, Aβ42 also showed a significant inverse association with MDD diagnosis (Exp(B) = 0.576; 95% CI: 0.350–0.950; *p* = 0.031). Specific data from each model are available in the Supplementary Material in Table S4.

### 2.4. Correlation Between Plasma Levels of Aβ40 and Aβ42 with Blood Parameters in MDD Patients

Significant inverse correlations were observed between erythrocyte counts and plasma amyloid-β levels in MDD patients. Therefore, higher erythrocyte counts were associated with lower concentrations of both Aβ40 and Aβ42 (Table 2). In contrast, no significant associations were found between plasma Aβ40 or Aβ42 levels and monocyte, neutrophil, or platelet counts. Similarly, biochemical parameters, including albumin, creatinine, and the AST/ALT ratio, did not show significant correlations with either amyloid-β peptide (Table 2).

### 2.5. Correlation Between Psychometric and Cognitive Scales and Plasma Levels of NfL, GFAP, and Aβ40 and Aβ42 in MDD Patients

Table 3 shows the values of both psychometric (HDRS, SAAS) and cognitive (MMSE, FCSRT) tests assessed in MDD patients with their respective subscales.

First, we observed a significant negative correlation between depression severity, as measured by the Hamilton Depression Rating Scale (HDRS), and circulating levels of both NfL (r = −0.372, *p* = 0.030) and GFAP (r = −0.351, *p* = 0.039).Expanded data can be consulted in the Supplementary Material in Table S5.

Depression severity was not significantly associated with age (r = −0.053, *p* = 0.763), and no significant differences in HDRS scores were found between male and female patients (U = 126.5, *p* = 0.699). When analyses were stratified by sex, the negative association between HDRS scores and NfL levels was no longer significant in either males (r = −0.414, *p* = 0.181) or females (r = −0.370, *p* = 0.082). In contrast, the association between HDRS scores and GFAP levels remained significant in males (r = −0.632, *p* = 0.031) but not in females (r = −0.209, *p* = 0.337). Second, GFAP levels were significantly and inversely correlated with identification, total recall, and delayed total recall scores, indicating an association between higher GFAP concentrations and poorer memory performance (Table 4).

**Figure 4:**
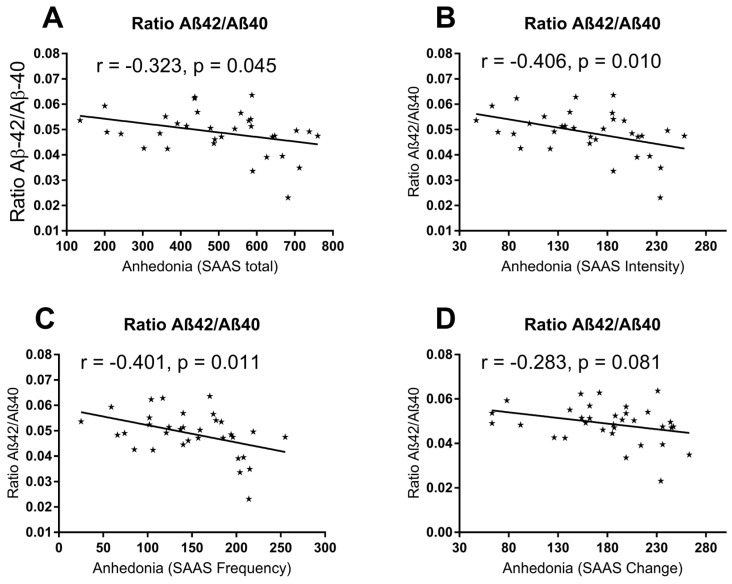
**Graphical associations between the Aβ42/Aβ40 ratio and anhedonia dimensions in patients with major depressive disorder.** Scatter plots illustrate the associations between the plasma Aβ42/Aβ40 ratio and (**A**) total anhedonia scores, as well as the (**B**) intensity, (**C**) frequency, and (**D**) change dimensions of anhedonia assessed using the Self-Assessment Anhedonia Scale (SAAS). Significant negative correlations were observed for total anhedonia, intensity, and frequency, whereas no significant association was found for the change dimension. Solid lines represent the best-fit regression lines. Correlation analyses were performed using Spearman’s rank correlation test.

Third, the Aβ42/Aβ40 ratio was significantly and negatively associated with total anhedonia scores, as well as with anhedonia intensity and frequency, but not with change over time (Figure 4). Total anhedonia scores measured by the Self-Assessment Anhedonia Scale (SAAS) were not significantly correlated with age (r = 0.121, *p* = 0.496), and no significant differences were observed between male and female patients with MDD (U = 127, *p* = 0.873). Detailed data on subjective scales can be checked in the Supplementary Material in Table S5 for the correlation between scales and markers.

Overall, our results indicate that patients with MDD exhibit a distinct peripheral biomarker profile characterized by reduced plasma amyloid-β peptides, whereas markers of neuroaxonal damage (NfL) and astroglial activity (GFAP) do not show consistent differences compared with HC.

Age emerged as a major determining factor of circulating NfL and GFAP levels, while amyloid-β peptides were not influenced by age. Reduced amyloid-β measures, particularly the Aβ42/Aβ40 ratio, were robustly associated with greater anhedonia severity, even after analyzing the potential influence of age and sex. In contrast, inverse associations between NfL or GFAP and depressive symptom severity (HDRS) were observed; however, these findings do not align with current biological hypotheses and could not be generally explained by age or sex, suggesting the potential influence of other unmeasured confounding factors, such as pharmacological treatment or clinical characteristics.

## 3. Discussion

In this work, we have simultaneously quantified plasma concentrations of NfL, GFAP, Aβ40, and Aβ42 in a small Spanish cohort of MDD patients and a healthy control (HC) group for the first time by using the ultrasensitive SIMOA platform. This fourth-generation approach represents a major methodological advancement over conventional immunoassays, allowing for simultaneous detection of subtle peripheral alterations and a precise picture of circulating markers in the range of picograms per milliliter (pg/mL) [33,34].

In our study, plasma NfL and GFAP levels showed no significant association with the MDD diagnosis, whereas both Aβ40 and Aβ42 were found to be significantly decreased in MDD patients but in the absence of a significant alteration of the Aβ42/Aβ40 ratio after adjusting for age and sex.

To the best of our knowledge, only one previous study has simultaneously measured these four biomarkers in MDD patients by using immunomagnetic reduction (IMR). Consistent with our findings, no significant differences were observed in plasma NfL or GFAP levels between MDD patients and controls. In contrast, no significant differences in Aβ40, Aβ42, or the Aβ42/Aβ40 ratio were found between groups [35]. To some extent, discrepant plasma Aβ40 and Aβ42 results across both studies may reflect differences in the analytical principles and assay design between platforms, including antibody configuration and epitope accessibility, which can influence the detection of distinct Aβ species [36].

In recent years, alterations in amyloid-β metabolism, particularly involving changes in Aβ40, Aβ42, and their relative balance (Aβ42/Aβ40 ratio), have been proposed as a potential biological mechanism contributing to the transition from depression to dementia [37,38,39,40]. To some extent, our data may support this hypothesis, although they are limited in their potential to represent changes at central levels. Alternatively, our pattern of results does not entirely resemble the classical Alzheimer’s disease (AD) profile, in which selective reductions in Aβ42 are accompanied by significant reductions in the Aβ42/Aβ40 ratio, which are considered critical indicators of cerebral amyloid deposition [41,42,43].

In line with this, our observed parallel reductions in amyloid-β peptides in MDD could be reflecting peripheral rather than central alterations. One plausible explanation relates to the intrinsic limitations of measuring brain-derived proteins in blood. Only a small fraction of cerebral proteins enters peripheral circulation, where they are further diluted by the substantially higher total protein concentration of plasma compared with cerebrospinal fluid (CSF), often by several orders of magnitude [44]. Nonetheless, the use of single-molecule array (SIMOA) technology partially mitigates these analytical constraints, as it enables the reliable detection of amyloid-β peptides and reduces the likelihood that the observed reductions simply reflect technical detection issues [45].

However, there are many other confounding factors that could explain the reduction of plasma Aβ peptides without necessarily reflecting decreased production at central levels. Briefly, circulating Aβ concentrations result from a dynamic balance between brain-derived efflux, peripheral production, and multiple clearance mechanisms. Therefore, alterations at some level could alter our interpretations. The transport of Aβ from the brain to the periphery is mediated by the blood–brain barrier (BBB) through specific transporters, such as Low-Density Lipoprotein Receptor-Related Protein 1 (LRP1) and P-glycoprotein, and may also involve non-canonical pathways, such as glymphatic and meningeal lymphatic pathways. Once in circulation, Aβ is subjected to rapid peripheral clearance through enzymatic degradation by proteases, including neprilysin, insulin-degrading enzyme, angiotensin-converting enzyme, and matrix metalloproteinases, as well as potential cellular uptake by monocytes, macrophages, and erythrocytes. In addition, peripheral organs, particularly the liver and the kidney, play a major role in Aβ removal, with hepatic clearance via soluble LRP1 representing a dominant elimination route. Moreover, systemic factors, such as inflammation, metabolic status, and vascular function, may further modulate these processes. Consequently, enhanced peripheral degradation or clearance, altered brain-to-blood transport, or systemic regulatory changes could lead to reduced plasma Aβ40 and Aβ42 levels without implying a corresponding decrease in central amyloid dynamics [32,46]. Other sources of peripheral Aβ, such as platelets, may contribute to circulating Aβ levels and weaken the abovementioned association between plasma and cerebrospinal fluid (CSF) concentrations, complicating the interpretation of peripheral Aβ as an incipient direct marker of central amyloid pathology [47].

Our exploratory analyses revealed an inverse association between erythrocyte counts and plasma Aβ40 and Aβ42 levels in MDD patients, whereas no significant correlations were observed for other blood cell populations. This finding should be interpreted with caution, as the analysis was restricted to the MDD group and could not be contrasted with HC, limiting its generalizability. In contrast, no associations were found between plasma Aβ levels and creatinine or the AST/ALT ratio, suggesting that the observed peripheral amyloid-β alterations are unlikely to be driven by overt renal or hepatic dysfunction. Overall, these results support a contribution of peripheral regulatory factors to plasma Aβ levels rather than a direct reflection of central amyloid pathology.

Moreover, we cannot exclude the interference of other confounding factors, such as body mass index (BMI), pharmacological treatment, or lipidic or metabolic alterations. Moreover, MDD is a well-known heterogeneous psychiatric disorder, and data regarding illness duration or the number of previous major depressive episodes would have strengthened the present analysis. Prior pharmacological treatment may have also modulated peripheral amyloid-β peptides. Relatedly, a previous study reported that reductions in peripheral Aβ42 were associated with depression, whereas decreases in plasma Aβ40 were influenced by pharmacological treatment [48]. However, these interpretations remain speculative and need further investigation.

Conversely, a recent meta-analysis of five studies reported no significant difference in Aβ plasma levels in MDD. Notably, studies including cognitively impaired individuals showed a modest positive association, suggesting that depressive symptoms may emerge during prodromal stages of dementia when amyloid pathology is already present [49]. Nevertheless, only two out of the five studies previously mentioned relied on formal DSM-IV MDD diagnosis. The first found no significant differences for Aβ levels in the plasma of MDD patients or their ratio when compared with controls, except for the group with mild cognitive impairment [50]. Conversely, the second study reported a significant reduction in the Aβ42/Aβ40 ratio in MDD patients, with baseline depressive severity predicting lower ratios independently of the cognitive state [51]. The other three studies assessed depressive symptomatology through questionnaires, indicating that their conclusions reflected a broader relationship between amyloid-β metabolism and depressive features in a late-life depressive context, rather than MDD diagnosis itself [48,52,53].

In our case, MDD patients were not cognitively affected as measured by MMSE, and no significant correlation was found between Aβ levels and depression severity. Nonetheless, a negative association between the Aβ42/Aβ40 ratio and anhedonia symptomatology were observed for intensity, frequency, and total scores. Although the absence of group differences in the Aβ42/Aβ40 ratio in MDD argues against an Alzheimer-like amyloid profile [54,55], the correlation with anhedonia was not causally associated with age or sex distribution, meaning that other parameters could have interfered.

On the other hand, GFAP and NfL levels have been simultaneously measured in patients with MDD, providing insights into potential astroglia and neuroaxonal damage [56,57]. One study found that only GFAP, but not NfL, was significantly increased in MDD patients, establishing a cut-off value of 128 pg/mL. However, age interferences cannot be excluded [58]. Another study found that only GFAP was significantly increased in the same biobank cohort. However, NfL was most robust in differentiating frontotemporal dementia from primary psychiatric disorders, such as MDD, schizophrenia, or bipolar disorder. It is important to note that both markers were not measured simultaneously in these cases [59,60].

Contradictorily, GFAP was found to be significantly lower after adjusting for age, sex, and BMI in MDD patients who have been drug-free for at least 2 months. Moreover, only NfL significantly increased after 3 months of aerobic exercise without pharmacological intervention, while GFAP remained stable [61]. Moreover, no baseline differences for plasma NfL and GFAP levels have been found between individuals with neuropsychiatric symptoms, including depression or anxiety, and controls. However, higher levels of GFAP and NfL predicted a future incidence and worsening of the symptoms prospectively [62]. More recently, NfL levels, but not GFAP, were found to be significantly higher in MDD patients [63], whereas others reported that both NfL and GFAP were significantly higher, proposing the GFAP/NfL ratio as a useful signature to distinguish MDD from Alzheimer’s disease. Nonetheless, sample size clearly limits the findings, and future research is needed [64].

The separate evaluation of GFAP and NfL also led to heterogeneous results. Our previous study [65] and others found no significant difference in NfL in MDD patients and controls [60,65,66,67,68,69], while a similar amount of other studies have reported a significant elevation of NfL in MDD patients [70,71,72,73,74]. Notoriously, significantly higher serum NfL levels were found in suicide attempters, from which 22 out of 50 had a diagnosis of MDD [75]. To the best of our knowledge, no study has yet found significantly decreased NfL levels in MDD patients, which either indicates a publication bias or that neuroaxonal damage might initiate in MDD but without being drastic, therefore not reaching statistical significance in the periphery. To some extent, age seems to be a strong factor, as was found in our study when separating by age ranges and the previous cut-off established by Rodero-Romero et al. for healthy populations [30].

Fewer studies have evaluated GFAP alone. Recently, significantly higher plasma GFAP concentrations were found in unmedicated MDD patients compared to controls after adjusting for age, sex, and BMI [76]. Similarly, another study found increased GFAP plasma levels in both adults and teenagers with MDD. Notably, GFAP levels significantly decreased after two weeks of antidepressant monotherapy with either selective serotonin reuptake inhibitors or serotonin–norepinephrine reuptake inhibitors [77]. Another study found a similar reduction in GFAP levels after 4 weeks with sertraline [78]. These results potentially suggest reversible astrocytic activation and a response marker for drug treatment in MDD.

In our study, both NfL and GFAP showed significant inverse associations with depression severity. Importantly, this pattern is inconsistent with prevailing biological hypotheses, which would predict higher levels of neuroaxonal and astroglial injury markers in more severe depressive states. As age may not account for these associations in our analyses, we consider that sex or unmeasured confounding factors may underlie these findings. In particular, pharmacological treatment or illness years represent plausible contributors, as patients with more severe or long-standing depression are more likely to have received intensive or prolonged treatment. Therefore, these results should be interpreted with caution. Only a few studies have found a significant correlation between NfL [71,73] or GFAP [58,76,77] and the HDRS scale, being in all cases positive associations rather than negative, suggesting that higher levels correlate with a more depressive symptomatology. Nonetheless, the vast majority of studies did not find a significant association between NfL or GFAP and depressive symptoms [35,59,61,63,65,66,68,69].

Notably, no significant association was found between NfL and the general cognitive state (MMSE), anhedonia symptomatology (SAAS), or episodic memory (FCSRT). In contrast, in AD and related disorders, higher plasma NfL and GFAP levels are consistently associated with lower MMSE scores and faster cognitive decline [79,80,81,82]. However, GFAP significantly and inversely correlated with identification (ID), total recall (TR) and delayed total recall (DTR) from the FCSRT test in the MDD patients, suggesting that increased astroglia activation may be linked to subtle impairments in episodic memory processes, particularly those involving encoding and retrieval [83]. Given the fact that the FCSRT primarily assesses hippocampal-dependent episodic memory and that astrocytic dysfunction has been implicated in AD, future research exploring this association could provide valuable insight into the cognitive relevance of peripheral astroglia activation.

**Figure 5:**
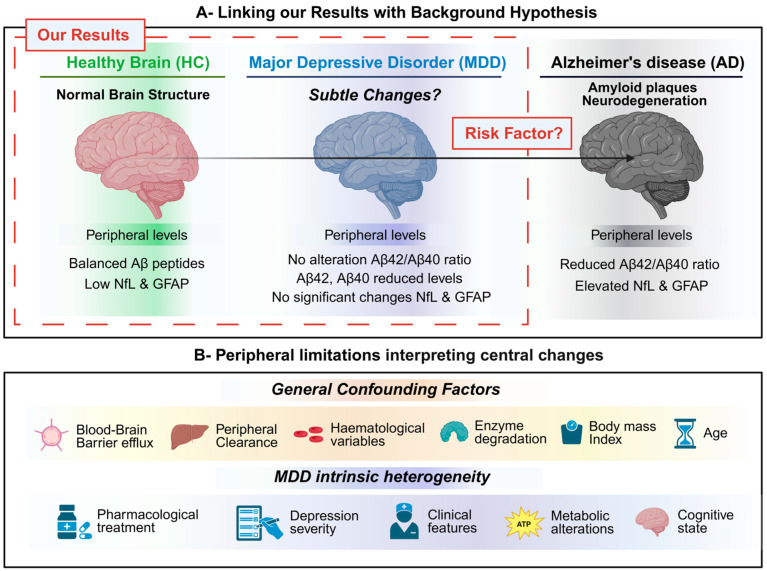
Peripheral biomarkers in major depressive disorder and Alzheimer’s disease: central hypotheses and interpretative limitations. (**A**) Conceptual framework linking peripheral biomarker findings with central pathology hypotheses. Healthy controls (HC) show preserved brain structure; major depressive disorder (MDD) is hypothesized to involve subtle brain alterations and act as a potential risk condition for neurodegeneration; and Alzheimer’s disease (AD) is characterized by amyloid plaques and neurodegeneration. Our results are based on peripheral measurements: HC show balanced Aβ peptides and low NfL and GFAP; MDD shows no change in the Aβ42/Aβ40 ratio, reduced Aβ42 and Aβ40 levels, and showed no significant changes in NfL and GFAP; AD shows a reduced Aβ42/Aβ40 ratio and increased NfL and GFAP. (**B**) Main biological and clinical factors limiting the interpretation of peripheral biomarkers as direct indicators of central nervous system pathology, including general confounders (e.g., blood–brain barrier transport, clearance, enzymatic degradation, BMI, age) and intrinsic heterogeneity of MDD (pharmacological treatment, symptom severity, clinical features, metabolic alterations, and cognitive state).

Overall, the present study aligns with the hypothesis that subtle biological alterations at the central level may emerge during MDD and potentially contribute to the increased risk of neurodegenerative conditions, such as Alzheimer’s disease. However, our findings are based exclusively on peripheral measurements of neurodegeneration-related biomarkers, whose circulating levels are influenced by multiple systemic and regulatory factors that complicate the interpretation of these subtle changes. Within this context, our results suggest that reductions in plasma amyloid-β peptides in MDD are more likely to reflect peripheral regulatory mechanisms rather than a classical Alzheimer’s disease-like neurodegenerative profile, particularly in the absence of concomitant changes in NfL and GFAP. These observations underscore the importance of cautious interpretations of peripheral biomarkers in psychiatric populations and highlight the need for integrative approaches combining peripheral and central measures in future studies (Figure 5).

Limitations and future perspectives. Despite being a pioneering study in Spain, several limitations should be acknowledged. First, the statistical power may have been limited by the relatively modest sample size, although it is comparable to that of contemporary case-control studies in the field. Second, cognitive assessments, such as MMSE and FCSRT, were not available for healthy controls (HC), which did not allow for direct group comparisons for these measures between MDD and HC or as covariates in the statistical models. These scales were assessed exclusively in MDD patients as part of routine clinical practice. Third, body mass index (BMI) could not be included as a covariate in the statistical models. Fourth, the potential influence of pharmacological treatment could not be adequately assessed. Consequently, pharmacological effects may have influenced peripheral biomarker levels. Finally, and most importantly, peripheral alterations do not necessarily reflect brain changes.

From our perspective, future studies should focus on these limitations by including larger and better-characterized cohorts, comprehensive cognitive assessments in control groups, and more detailed data on clinical and metabolic confounders. Such approaches will be essential to further evaluate the potential of SIMOA-based peripheral biomarkers (e.g., NfL, GFAP, Aβ40, and Aβ42) as tools to detect subtle biological alterations associated with MDD and to explore their possible role as early indicators or monitoring biomarkers of cognitive decline. In this context, and considering the established link between a psychiatric disorder such as MDD and an increased risk of Alzheimer’s disease, longitudinal studies will be required to determine whether these peripheral biomarkers may contribute to the early identification of individuals at higher risk of subsequent neurodegenerative processes.

## 4. Materials and Methods

### 4.1. MDD Patients and HC Controls

We introduce a cross-sectional observational study that began in February 2019 with the recruitment process and ended in February 2025 with the experimental procedure. The recruitment process finished in March 2020 due to the beginning of COVID-19 in Spain. Samples were then stored at ultra-freezing conditions (−80 °C) until the acquisition of the SIMOA technology in February 2024. We recruited 35 patients who met the DSM-5 diagnostic criteria for major depressive disorder (MDD group, n = 35) at the Álvaro Cunqueiro Hospital (Vigo, Spain). We also took samples from 32 volunteers who were included as the healthy control group (HC group, n = 32).

The inclusion criteria for patients were meeting the DSM-5 criteria for major depressive disorder and being adults according to Spanish national legislation (age ≥ 18 years). The exclusion criteria included suffering from additional comorbidities, such as neurological pathologies, cancer, or cardiovascular diseases. Women who were pregnant or in the lactation period were also excluded from the study. In the HC group, the same exclusion criteria were applied, with the additional exclusion of individuals with any history of psychiatric disorders or use of psychiatric medication.

All MDD patients and HC volunteers were of Spanish nationality. This research was performed according to the Declaration of Helsinki. We obtained written consent from all participants or their legal guardians if this was considered necessary.

### 4.2. Blood Sample Extraction

Blood samples were extracted under fasting conditions in the morning between 7:00 and 10:00 am at the Álvaro Cunqueiro Hospital between 2019 and 2020 from the antecubital portion of the arm and stored in EDTA tubes. Plasma was then immediately separated through differential centrifugation (2000 rpm, 35 min). We then stored aliquots in ultra-low freezing conditions (−80 °C) until protein measurement at the Health Research Institute of Santiago de Compostela (IDIS).

### 4.3. Blood Measure Parameters

Peripheral blood parameters, including erythrocyte, platelet, monocyte, and neutrophil counts, as well as biochemical markers, such as creatinine and liver enzymes, were obtained as part of routine clinical assessment. Blood samples were collected on the same day as plasma separation for biomarker determination and analyzed using standard hematological and biochemical methods at the Hospital Álvaro Cunqueiro (Vigo, Spain). Aspartate aminotransferase (AST), alanine aminotransferase (ALT), and the AST/ALT ratio were used as indicators of hepatic function, while creatinine was considered a marker of renal function.

### 4.4. Ultrasensitive Protein Measurement

Plasma samples were moved to Santiago de Compostela in dry ice and defrosted at room temperature, therefore being subjected to a single freeze–thaw cycle, and centrifugated at 10,000 RCF for 5 min according to the manufacturer’s instructions. Then, 100 µL of plasma was pipetted in each well of a 96-well plate. We used the Platform for Biomarkers in Biological Fluids (SIMOA^®^ HD-X) available at the Health Research Institute of Santiago de Compostela (IDIS). Specifically, we used the SIMOA^®^ Neurology 4-Plex E Advantage PLUS kit (QuanterixTM Corp, Billerca, MA, USA) to measure NfL, GFAP, Aβ42, and Aβ40 plasma levels. We made two replicates of each sample with intra-assay CVs of 6.01 ± 4.66%, 3.84 ± 2.57%, 4.43 ± 5.98%, and 1.85 ± 1.44%, respectively.

### 4.5. Psychometric and Cognitive Scales

Psychometric scales were administered to MDD patients by trained clinicians. Depression severity was assessed by using the Hamilton Depression Rating Scale (HDRS) [84]. Anhedonia symptomatology was measured with the Self-Assessment Anhedonia Scale (SAAS) [85]. The general cognitive state was evaluated with the Mini-Mental State Examination (MMSE) [86]. Finally, episodic memory was assessed using the Free and Cued Selective Reminding Test (FCSRT) [87].

### 4.6. Statistical Analysis

Continuous variables are presented as mean ± standard deviation. Normality was assessed using the Shapiro–Wilk test for quantitative variables (NfL, GFAP, Aβ-40, Aβ-42, psychometric and cognitive scales, and age). Homogeneity of variances was evaluated using Levene’s test. When variables were normally distributed (*p* > 0.05) and showed homogeneity of variances (*p* > 0.05), between-group comparisons were performed using Student’s *t*-test. When normality or homoscedasticity assumptions were not followed, the non-parametric Mann–Whitney U test was applied. Associations between variables were examined using Spearman’s rank correlation coefficient (r_s_). Multivariable logistic regression analyses adjusted for age and sex were performed for each biomarker. Statistical analyses were conducted using GraphPad Prism (version 7.05) and IBM SPSS Statistics (version 29.0.1.0).

## 5. Conclusions

Here, we present the first cross-sectional observational study conducted in Spain to simultaneously evaluate plasma levels of NfL, GFAP, Aβ40, and Aβ42 in patients with major depressive disorder (MDD) compared with healthy controls and their associations with depressive symptom severity, anhedonia, and cognitive performance. After adjustment for age and sex, reduced plasma levels of Aβ40 and Aβ42 were consistently associated with MDD, whereas NfL and GFAP showed no independent association.

NfL and GFAP were strongly influenced by age and showed limited disease specificity, suggesting that these markers may primarily reflect age-related neuroaxonal and astroglial processes rather than core depressive pathology. In contrast, reductions in amyloid-β peptide, particularly their association with anhedonia severity, even in the absence of incipient cognitive impairment, suggest a potential line of research. Nonetheless, these findings should be interpreted with caution, as metabolic factors or treatment-related effects may contribute to peripheral amyloid-β alterations and no causal inference can be inferred.

Overall, our results support the relevance of SIMOA for detecting subtle peripheral alterations in MDD while highlighting the need for cautious interpretation of neurodegeneration-related markers in psychiatry. Longitudinal studies will help to study whether peripheral amyloid-β reductions contribute to later neurodegenerative risk and their potential utility for patient stratification in MDD.

## Figures and Tables

**Table 1:** Comparison between healthy controls (HC) and patients with major depressive disorder (MDD).

Variables	HC (N = 32)	MDD (N = 35)	*p*-Value
Sex (F/M)	13/19	23/12	0.040 ^a^
Age (years)	40.97 ± 13.37	47.80 ± 11.12	0.026 ^b^
NfL (pg/mL)	6.10 ± 2.89	7.65 ± 3.69	0.088 ^c^
GFAP (pg/mL)	39.61 ± 13.94	48.44 ± 22.16	0.112 ^c^
Aβ40 (pg/mL)	77.61 ± 22.57	64.55 ± 23.52	0.028 ^c^
Aβ42 (pg/mL)	3.92 ± 1.31	3.20 ± 1.05	0.004 ^c^
Ratio Aβ42/Aβ40	0.0519 ± 0.0156	0.0490 ± 0.0085	0.184 ^c^

Statistical comparisons were made with ^a^ Chi-square test, ^b^ Student’s *t*-test and, ^c^ U Mann–Whitney test. F: female, M: male. NfL: neurofilament light chain, GFAP: glial fibrillary acidic protein, Aβ: amyloid beta peptides. Ratio: calculated as the direct division of Aβ42 by Aβ40 levels.

**Table 2:** Correlations between plasma amyloid-β peptides and selected blood parameters in patients with major depressive disorder (MDD).

Blood Parameters	Mean ± SD	Range	Aβ40 (pg/mL)		Aβ42 (pg/mL)	
			r	*p*	r	*p*
Erythrocytes (10^6^/µL)	4.56 ± 0.60	[4.0–5.2]	−0.416	0.022	−0.533	0.002
Monocytes (10^3^/µL)	0.39 ± 0.07	[0.2–1.0]	0.150	0.428	0.159	0.401
Neutrophils (10^3^/µL)	3.75 ± 1.33	[1.5–7.5]	−0.132	0.486	−0.069	0.718
Platelets (10^3^/µL)	232.67 ± 76.56	[130.0–450.0]	0.059	0.758	0.230	0.221
Albumin (g/dL)	3.98 ± 0.45	[3.4–5.0]	−0.188	0.321	−0.208	0.271
Creatinine (mg/dL)	0.72 ± 0.12	[0.5–1.1]	−0.039	0.837	−0.176	0.353
AST/ALT	0.9 ± 0.39	[0.7–1.3]	0.205	0.286	0.139	0.472

Values are expressed as mean ± standard deviation (SD). Ranges indicate the standard physiologically healthy range for each blood parameter. Correlation coefficients (r) and corresponding *p* values were obtained using Spearman’s rank correlation analysis. Amyloid-β 40 (Aβ40) and amyloid-β 42 (Aβ42) plasma concentrations are expressed in pg/mL. Erythrocytes, monocytes, neutrophils, and platelets are expressed as absolute counts (10^6^/µL or 10^3^/µL, as indicated). Albumin and creatinine concentrations are expressed in g/dL and mg/dL, respectively. The AST/ALT ratio represents the aspartate aminotransferase to alanine aminotransferase ratio.

**Table 3:** Psychometric scales in patients with major depressive disorder (MDD).

Psychometric Scales		Mean ± SD
HDRS		22.94 ± 5.96
MMSE		23.83 ± 6.87
	Intensity	160.6 ± 55.9
	Frequency	150.0 ± 54.1
SAAS	Change	182.4 ± 53.7
	Total	502.7 ± 165.1
	Identification	15.12 ± 1.09
	TFR	33.73 ± 7.22
FCSRT	TR	38.62 ± 7.45
	DFR	12.60 ± 2.61
	DTR	14.67 ± 1.58

Mean values and the standard deviation (SD) of each psychometric scale are shown. HDRS: Hamilton Depressive Rating Scale; MMSE: Mini-mental State Examination; SAAS: Self-Assessment Anhedonia Scale; FCSRT: Facilitated and Cued Selective Reminding Test; TFR: total free recall; TR: total recall; DFR: delayed free recall; DTR: delayed total recall.

**Table 4:** Correlation between peripheral markers and episodic memory assessed by the FCSRT test.

Memory Scale		NfL		GFAP		Aβ40		Aβ42	
		(pg/mL)		(pg/mL)		(pg/mL)		(pg/mL)	
		r	*p*	r	*p*	r	*p*	r	*p*
FCSRT	ID	0.011	0.951	−0.385 *	0.012	−0.162	0.306	−0.176	0.271
	TFR	0.159	0.370	−0.275	0.078	−0.251	0.109	−0.114	0.478
	TR	0.028	0.874	−0.358 *	0.020	−0.177	0.262	−0.178	0.266
	DFR	0.226	0.200	−0.177	0.262	−0.097	0.543	0.082	0.610
	DTR	−0.054	0.761	−0.339 *	0.028	−0.243	0.121	−0.102	0.524

Correlation analysis was performed with Spearman’s correlation coefficient. FCSRT: Facilitated and Cued Selective Reminding Test. TFR: total free recall; TR: total recall; DFR: delayed free recall; DTR: delayed total recall. NfL: neurofilament light chain; GFAP: glial fibrillary acidic protein; Aβ40: amyloid-β 1–40; Aβ42: amyloid-β 1–42. * indicates significant *p*-values (*p* < 0.05).

## Data Availability

The original contributions presented in this study are included in the article/Supplementary Material. Further inquiries can be directed to the corresponding authors.

## Supplementary Materials

The following supporting information can be downloaded at https://www.mdpi.com/article/10.3390/ijms27031474/s1.

## Abbreviations

The following abbreviations are used in this manuscript:

Aβ	Amyloid-β peptides
AD	Alzheimer’s disease
CSF	Cerebrospinal fluid
FCSRT	Free and Cued Selective Reminding Test
GFAP	Glial fibrillary acidic protein
HDRS	Hamilton Depression Rating Scale
MDD	Major depressive disorder
MMSE	Mini-Mental State Examination
NfL	Neurofilament light chain
SAAS	Self-Assessment Anhedonia Scale
SIMOA	Single-molecule array
