# Supplementary material for: Reduced Plasma Aβ Peptides but Stable NfL and GFAP in Major Depressive Disorder
Source: Int J Mol Sci. 2026 Feb 2;27(3):1474. doi: 10.3390/ijms27031474 (PMC12898547; doi:10.3390/ijms27031474)
Supplement: Supplementary file 1 [file ijms-27-01474-s001.zip › ijms-4083200-supplementary.pdf]

Supplementary material

Table S1. Demographic and clinical characteristics and peripheral biomarker levels across age ranges.

	Age ranges for Figure 2 (years)				
	21-30	31-40	41-50	51-60	61-70
**Sample size (N)**	12	12	21	13	9
**Age (Years)**	26 ± 2.4	35.8 ± 3.1	44.9 ± 1.3	55.9 ± 2.8	63.8 ± 2.3
**Sex (M/F)**	8/4	2/10	10/11	7/6	4/5
**Group (HC/MDD)**	9/3	8/4	7/14	3/10	5/4
**NfL (pg/ml)**	4.54 ± 1.18	4.57 ± 1.41	6.57 ± 2.65	8.68 ± 2.69	11.53 ± 3.96
**GFAP (pg/ml)**	34.58 ± 13.61	40.18 ± 14.24	40.48 ± 13.04	48.03 ± 23.32	65.72 ± 22.01
**Aβ40 (pg/ml)**	69.44 ± 24.15	77.32 ± 14.11	67.18 ± 25.42	74.50 ± 25.91	66.92 ± 29.09
**Aβ42 (pg/ml)**	3.81 ± 1.51	3.65 ± 1.13	3.47 ± 1.38	3.62 ± 0.72	3.21 ± 1.28
**Ratio Aβ42/Aβ40**	0.0531 ± 0.0103	0.0484 ± 0.0146	0.0516 ± 0.0062	0.0464 ± 0.0087	0.0520 ± 0.0239

Data are presented across five age ranges (21–30, 31–40, 41–50, 51–60, and 61–70 years). Values are expressed as mean ± standard deviation. Sample size (N), age (years), sex distribution (male/female), and diagnostic group: healthy controls (HC) and major depressive disorder (MDD) are shown for each age range. Plasma levels of neurofilament light chain (NfL), glial fibrillary acidic protein (GFAP), amyloid-β 40 (Aβ40), amyloid-β 42 (Aβ42), and the Aβ42/Aβ40 ratio are reported in pg/ml.

Table S2. Demographic and clinical characteristics and peripheral biomarker levels according to age cut-offs.

**Markers**	Age ranges for Figure 2 (years)			
	≤ 45	> 45	< 55	≥ 55
**Sample size (N)**	37	30	48	19
**Age (Years)**	35.2 ± 7.5	56.1 ± 6.6	38.3 ± 8.8	60.3 ± 3.9
**Sex (M/F)**	17/20	14/16	22/26	9/10
**Group (HC/MDD)**	22/15	10/20	25/23	7/12
**NfL (pg/ml)**	5.26 ± 1.95	8.98 ± 3.72	5.60 ± 2.27	10.11 ± 3.62
**GFAP (pg/ml)**	37.30 ± 12.70	52.76 ± 22.17	39.37 ± 13.62	56.50 ± 25.08
**Aβ40 (pg/ml)**	71.72 ± 22.33	69.64 ± 25.90	70.88 ± 22.50	70.55 ± 27.57
**Aβ42 (pg/ml)**	3.70 ± 1.35	3.37 ± 1.04	3.59 ± 1.31	3.46 ± 1.02
**Ratio Aβ42/Aβ40**	0.0515 ± 0.0108	0.0490 ± 0.0143	0.0507 ± 0.0099	0.0496 ± 0.0179

Participants were stratified according to two age cut-offs (≤45 vs. >45 years, and <55 vs. ≥55 years) to evaluate age-related differences in peripheral biomarker levels. Data are presented as mean ± standard deviation. Sample size (N), age, sex distribution (male/female), and diagnostic group: healthy controls (HC) and major depressive disorder (MDD) are shown for each subgroup. Plasma concentrations of neurofilament light chain (NfL), glial fibrillary acidic protein (GFAP), amyloid-β 40 (Aβ40), amyloid-β 42 (Aβ42), and the Aβ42/Aβ40 ratio are reported in pg/ml.

Table S3. Peripheral biomarker levels and clinical, affective, and cognitive measures stratified by sex.

Markers		Female (n = 36)	Male (n = 31)	p-value
Age (years)		44.42 ± 11.76	44.68 ± 13.77	0.934
Group (HC/MDD)		13/23	23/8	-
NfL (pg/ml)		7.36 ± 3.77	6.37 ± 2.88	0.418
GFAP (pg/ml)		50.85 ± 21.11	36.53 ± 12.94	0.002
Aβ40 (pg/ml)		72.74 ± 20.86	68.51 ± 27.04	0.546
Aβ42 (pg/ml)		3.47 ± 1.13	3.66 ± 1.35	0.562
Ratio Aβ42/Aβ40		0.0485 ± 0.0109	0.0527 ± 0.0138	0.395
HDRS		22.5 ± 5.5	23.8 ± 6.9	0.699
MMSE		22.5 ± 7.0	26.4 ± 6.0	0.279
SAAS	Intensity	158.8 ± 53.0	163.7 ± 63.2	0.817
	Frequency	148.3 ± 52.3	153.1 ± 59.5	0.866
	Change	179.7± 53.8	187.2 ± 55.6	0.729
	Total	497.9± 166.3	511.6 ± 14.9	0.873
FCSRT	ID	15.3 ± 1.1	14.9 ± 1.2	0.311
	TFR	35.1 ± 6.5	31.1 ± 8.1	0.158
	TR	39.7 ± 7.4	36.5 ± 7.5	0.098
	DFR	13.0 ± 2.3	11.7 ± 3.0	0.187
	DTR	14.8 ± 1.2	14.5 ± 2.2	0.974

Data are presented separately for female and male participants. Values are expressed as mean ± standard deviation. Group distribution: healthy controls (HC) and major depressive disorder (MDD), plasma levels of neurofilament light chain (NfL), glial fibrillary acidic protein (GFAP), amyloid-β 40 (Aβ40), amyloid-β 42 (Aβ42), and the Aβ42/Aβ40 ratio are shown, together with clinical: Hamilton Depressive Rating Scale (HDRS), Self-Assessment Anhedonia Scale (SAAS) as total scores and separated by intensity, frequency, change and cognitive measures: Mini-Mental State Examination (MMSE) and the Free and Cued Selective Reminding Test (FCSRT) and its subscales of identification (ID), total free recall (TFR), total recall (TR), delayed free recall (DFR), and delayed total recall (DTR). Between-group comparisons were performed using the Mann–Whitney U test, and corresponding p-values are reported.

Table S4. Multivariate logistic regression models adjusted for age and sex were used to assess the association between each biomarker and diagnostic group (MDD vs HC).

NfL<br>Model	B	SE	Wald	Df	p	Exp(B)	95% C.I. para EXP(B)	
							Inferior	Superior
Sex	1.077	0.548	3.867	1	0.049	2.935	1.004	8.586
Age	0.045	0.029	2.459	1	0.117	1.046	0.989	1.106
NfL	0.025	0.108	0.053	1	0.818	1.025	0.830	1.267
Constant	-2.669	1.116	5.714	1	0.017	0.069	-	-
GFAP<br>Model	B	SE	Wald	df	p	Exp(B)	95% C.I. para EXP(B)	
							Inferior	Superior
Sex	1.087	0.591	3.378	1	0.066	2.965	0.930	9.449
Age	0.048	0.025	3.655	1	0.056	1.049	0.999	1.101
GFAP	0.004	0.018	0.049	1	0.825	1.004	0.969	1.040
Constant	-2.788	1.127	6.125	1	0.013	0.062	-	-
Aβ40<br>Model	B	SE	Wald	df	p	Exp(B)	95% C.I. para EXP(B)	
							Inferior	Superior
Sex	1.418	0.590	5.786	1	0.016	4.130	1.300	13.117
Age	0.056	0.024	5.380	1	0.020	1.057	1.009	1.108
Abeta40	-0.034	0.014	5.928	1	0.015	0.967	0.941	0.993
Constant	-0.740	1.346	0.302	1	0.582	0.477	-	-
Aβ42<br>Model	B	SE	Wald	df	p	Exp(B)	95% C.I. para EXP(B)	
							Inferior	Superior
Sex	1.252	0.570	4.813	1	0.028	3.496	1.143	10.694
Age	0.047	0.023	4.033	1	0.045	1.048	1.001	1.097
Abeta42	-0.551	0.255	4.670	1	0.031	0.576	0.350	0.950
Constant	-0.723	1.394	0.270	1	0.604	0.485	-	-

Multivariate logistic regression models were performed to explore the association between plasma biomarkers and diagnostic group (MDD vs HC), adjusting for sex and age. In each model, B represents the regression coefficient (log-odds), SE the standard error of the coefficient, Wald the test statistic used to determine whether the coefficient significantly differs from zero, df the degrees of freedom, p-value the level of statistical significance (with $p < 0.05$ considered significant), and Exp(B) the odds ratio (OR), which indicates the change in odds of having MDD per unit increase in the predictor variable, accompanied by its 95% confidence interval (CI).

Table S5. Values of psychometric (HDRS, SAAS) and cognitive (MMSE, FCSRT) from MDD patients and their correlations with each plasma marker (NfL, GFAP, Aβ40 and Aβ42).

**Psychometric and Cognitive scales**		**Mean ± SD**	**NfL (pg/ml)**		**GFAP (pg/ml)**		**Aβ40 (pg/ml)**		**Aβ42 (pg/ml)**		**Ratio Aβ42/Aβ40**	
			**r**	**p**	**r**	**p**	**r**	**p**	**r**	**p**	**r**	**p**
**HDRS**		22.94±5.96	**-0.372***	**0.030**	**-0.351***	**0.039**	-0.268	0.120	-0.234	0.182	-0.230	0.191
**MMSE**		23.83±6.87	-0.312	0.073	0.029	0.869	-0.056	0.749	-0.088	0.620	-0.280	0.108
**SAAS**	**Intensity**	160,6±55.9	-0,071	0.696	0.016	0.921	0-.060	0.711	-0.192	0.241	**-0.406***	**0.010**
	**Frequency**	150,0±54.1	-0,058	0.748	-0.039	0.811	-0.095	0.560	-0.228	0.163	**-0.401***	**0.011**
	**Change**	182.4±53.7	-0,213	0.234	0.023	0.889	-0.167	0.303	-0.256	0.115	-0.283	0.081
	**Total**	502.7±165.1	-0,224	0.210	0.180	0.910	-0.131	0.421	-0.241	0.139	**-0.323***	**0.045**
**FCSRT**	**ID**	15.12±1.09	0,011	0.951	**-0.385***	**0.012**	-0.162	0.306	-0.176	0.271	-0.068	0.675
	**TFR**	33.73±7.22	0,159	0.370	-0.275	0.078	-0.251	0.109	-0.114	0.478	0.255	0.108
	**TR**	38.62±7.45	0,028	0.874	**-0.358***	**0.020**	-0.177	0.262	-0.178	0.266	0.033	0.839
	**DFR**	12.60±2.61	0,226	0.200	-0.177	0.262	-0.097	0.543	0.082	0.610	0.244	0.125
	**DTR**	14.67±1.58	-0,054	0.761	**-0.339***	**0.028**	-0.243	0.121	-0.102	0.524	0.061	0.704

The second column indicates the values from psychometric and cognitive scales obtained after assessing MDD patients as mean and standard deviation (SD). HDRS: Hamilton Depressive Rating Scale. MMSE: Mini-Mental State Examination. SAAS: Self-Assessment Anhedonia Scale. I: Intensity; F: Frequency and C: Change. Total is the arithmetic sum from each domain (Total = I+F+C). FCSRT: Facilitated and Cued Selective Reminding Test. ID: Identification. TFR: Total Free Recall. TR: Total Recall. DFR: Delayed Free Recall. DTR: Delayed Total Recall. Statistical correlation; r: Rho Spearman's correlation, p: p-value. NfL: neurofilament light chain, GFAP: glial fibrillary acidic protein, Aβ: amyloid beta peptides. Ratio: calculated as the direct division of Aβ42 by Aβ40 levels. *Indicates statistical significance (p-value < 0.05).
